# Vascularisation Pattern of Chronic Pancreatitis Compared with Pancreatic Carcinoma: Results from Contrast-Enhanced Endoscopic Ultrasound

**DOI:** 10.1155/2012/420787

**Published:** 2012-07-10

**Authors:** Michael Hocke, Christoph F. Dietrich

**Affiliations:** ^1^Department Internal Medicine II, Hospital Meiningen GmbH, Bergstrasse 3, 98617 Meiningen, Germany; ^2^Department of Internal Medicine II, Caritas Hospital Bad Mergentheim, Uhlandstraße 7, 97980 Bad Mergentheim, Germany

## Abstract

Discriminating between focal chronic pancreatitis and pancreatic cancer is always a challenge in clinical medicine. Contrast-enhanced endoscopic ultrasound using Doppler techniques can uniquely reveal different vascularisation patterns in pancreatic tissue alterated by chronic inflammatory processes and even allows a discrimination from pancreatic cancer. This paper will describe the basics of contrast-enhanced high mechanical index endoscopic ultrasound (CEHMI EUS) and contrast enhanced low mechanical index endoscopic ultrasound (CELMI EUS) and explain the pathophysiological differences of the vascularisation of chronic pancreatitis and pancreatic carcinoma. Furthermore it will discuss how to use these techniques in daily clinical practice.

## 1. Introduction

Adenocarcinoma of the pancreas is one of the solid carcinomas with the worst prognosis [1, 2]. The medium survival rate of an untreated pancreatic carcinoma is 6 months after the diagnosis is made [3]. Despite better diagnostic methods the detection of pancreatic cancer in an early stage is still a rare event [4]. This is due to the fact that the organ is not easy to investigate by percutaneous ultrasound and so far we do not have screening strategies even in high risk patients [5]. Even when diagnosed in time and treated by surgery the medium survival rate is 13.9 months [6]. The likely reason for the poor outcome of pancreatic cancer patients is the early micrometastatic spreading.

Unfortunately pancreatic carcinoma can mimic focal chronic pancreatitis. Because of the good resolution, modern diagnostic tools like endosonography, computed tomography (CT), or magnetic resonance imaging (MRI) can detect even small lesions in the pancreas down to 5 mm or less in size; however the discrimination of these lesions remains a challenge. This is due to the fact that even the imaging produced by contrast-enhanced CT [7] or MRI scanners [8] can be inconclusive. It is well known that adenocarcinoma of the pancreas normally shows less contrast enhancing effect than the surrounding pancreatic tissue and should be therefore possible to identify [9]. However in at least 10% of cases, these tumors have no visible contrast enhancing differences and remain hidden for CT and MRI scanners [7]. This effect can be even higher in patients with inflammatory pancreatic tissue. Chronic inflammation of the pancreas can lead to impaired contrast enhancing behavior of the normal tissue and can therefore hide the tumor. This is even more important because most adenocarcinomas (approx. 65%) are localized in the pancreatic head and lead to incomplete or complete pancreatic duct invasion with secondary chronic inflammation of the remaining pancreas [10]. In those cases the sensitivity and specificity of contrast-enhanced methods can go down to round about 70% (Table 1).

After introducing positron emissions tomography (PET) into clinical practice tumor diagnosis was supposed to be much more reliable. The advantage of using PET for diagnosis is the option of metabolic imaging of processes. However pancreatic carcinoma can have the same metabolic characteristics like chronic pancreatitis and therefore PET was also not able to produce reliable results [12, 13].

Histology is so far the only definitive diagnostic option for discrimination between pancreatic carcinoma and chronic pancreatitis. Because of sampling errors not even percutaneous biopsy of the pancreas is reliable and comes with the risk of cancer cell seeding [14]. This is the reason that preoperative biopsy of suspected pancreatic carcinoma is not recommended in recent guidelines like the German guideline for pancreatic carcinoma [15] or the international guideline for pancreatic cancer [16]. The guidelines recommend operative resection in every case of suspected pancreatic carcinoma which appears resectable. However this comes with an insignificant risk of morbidity and even mortality especially if the suspicion is not confirmed postoperatively and turns out to be a chronic pancreatitis [17, 18].

Intraoperative cytology seems to be an effective method to get the diagnosis [19] but still remains an invasive procedure. Endoscopic fine needle puncture of the lesion seems to be reliable in the absence of chronic pancreatitis [20]; however it cannot always provide reliable results in the presence of chronic pancreatitis again mostly because of sampling errors [21]. In addition even endoscopic fine needle cytology is not recommended per example by the German guidelines of pancreatic diseases because of the marginal risk of cell seeding and the catastrophic prognosis of the metastatic disease [22]. However there is increasing evidence that cell seeding is a rare phenomenon and due to the fact that the area of puncture will be removed by the operation is nearly neglectable [23], still it would be preferable to preselect patients for endoscopic fine needle puncture and furthermore the targeting area.

So far the problem of differential diagnosis of focal chronic pancreatitis and pancreatic carcinoma remains unsolved.

## 2. Contrast-Enhanced Ultrasound: A Step Forward in the Differential Diagnosis of Pancreatic Diseases

Contrast-enhanced ultrasound was already performed from 1982 mainly for echocardiographic reasons to enhance the echo signal [24]. In 1990 a first generation ultrasound contrast enhancer appeared for abdominal ultrasound [25]. After the first positive results in transcutanous ultrasound of the liver, contrast-enhanced ultrasound of the parenchymatous organs was born [26].

The gas bubbles of the contrast enhancers of the first generation were not stable enough for continuous ultrasound scanning. This meant that the sweep technique had to be developed. In this technique the contrast enhancer was injected and scanning was performed with high mechanical index after 2-3 minutes with a sweep over the suspected lesion. During the sweep the bubbles were destroyed and a contrast-enhanced image could be created normally with the help of the power Doppler mode. Especially for discrimination of liver lesions this method revealed astonishing results [27, 28].

The main disadvantage of this method was the scattered scanning of the lesion. Basically it was not different to CT and MRI scans with only one or two short chances to see the contrast enhancer effect and the lack of continuous scanning.

After a new contrast enhancer generation (SonoVue, Bracco) was introduced 2001/2002 [29] soon a new ultrasound technique was established. Ultrasound scanning with low mechanical index [30] could evolve [31]. These techniques allowed continuous ultrasound scanning of the contrast enhancer influx and distribution in the parenchymatous organs and therefore produced new insights into contrast enhancing dynamics.

The main advantage of the possibility of continuous scanning is the real-time viewing of the contrast enhancer effects.

Most studies were performed for liver lesions. This is due to the fact that the liver is fed by two different vessel systems (arterial blood and portal vein blood). Especially the portal vein system makes the differentiation of liver tissue-like lesions with portal veins inside (e.g., focal nodular hyperplasia) from metastatic tissue without portal veins inside (e.g., colonic cancer metastasis) easy [32, 33].

It has to be mentioned that the use of contrast-enhanced ultrasound is not approved for other parenchymatous organs than the liver. However lots of studies have already been done for basically all parenchymatous organs including the pancreas so that recently the European Federation of Societies for Ultrasound in Medicine and Biology (EFSUMB) published their guidelines for clinical practice [34].

Percutaneous contrast-enhanced ultrasound for pancreatic diseases is nowadays nearly as widespread in clinical practice as liver investigations [35–38]. Although percutaneous ultrasound has already an incredible resolution, it is sometimes hampered by overlying air or patient's physiognomy [39]. Using the contrast enhancing effect in endoscopic ultrasound was a logical progression, but the technique could not develop quickly because of the lack of low mechanical index high resolution ultrasound probes.

Some interesting studies however could show the feasibility of the contrast-enhanced endoscopic ultrasound in a color Doppler setting using high mechanical index ultrasound [40–42]. This was the beginning of a new understanding of the underlying processes which made it possible to give a new dimension to the differential diagnosis of chronic pancreatitis to pancreatic carcinoma.

## 3. Understanding the Neovascularisation of Chronic Pancreatitis and Pancreatic Carcinoma for Differential Diagnosis

Becker et al. could show that using ultrasound contrast enhancer for endoscopic ultrasound in a Doppler mode (Power Doppler mode) was able to reveal different enhancement patterns from chronic pancreatitis and pancreatic carcinoma [43]. In a preliminary study they assumed they had produced a contrast enhancing effect in the pancreatic tissue like the Levovist studies in the liver or contrast enhancing effects of CT or MRI scans. They were able to show a contrast enhancing effect in all patients studied with chronic pancreatitis and a lack of contrast enhancing effect in almost all patients with pancreatic carcinoma. The basic misunderstanding at that time was that using a Doppler technique in high mechanical index mode can only lead to a contrast enhancing effect in vessels but not in the pancreatic tissue. The tissue enhancing or nonenhancing effect was based on multiple microvessels combined with a blooming effect after contrast enhancer influx.

Understanding those basics led to the realisation that later on in the scanning process the underlying multiple microvessels could be imaged after the self-limitation of the blooming effect. Once the microvessels could be visualized two different vessel patterns appeared [44].

Typical for chronic pancreatitis is a netlike homogenous and rich microvessel system over the whole lesion. In contrast, pancreatic carcinoma shows an irregular and diminished microvessel system without a netlike appearance. It should be emphasized that those microvessel patterns could not be detected before the introduction of contrastenhanced endoscopic ultrasound. To visualize those vessels, a method with a high resolution has to be combined with a contrast technique of microvessels. CT and MRI scan as well as angiographic methods are not able to produce this kind of resolution. Doppler techniques alone even with high resolution ultrasound probes cannot provide the necessary effect to analyze those vessel systems either [45].

However, the knowledge of the different types of microvascularisation cannot discriminate chronic pancreatitis from pancreatic cancer in every case. It has to be taken into account that small cell adenocarcinomas of the pancreas with a rich vessel system and forms of chronic pancreatitis with abundant fibrous tissue and a diminished vessel system exist. Using the technique of contrast-enhanced endoscopic power Doppler ultrasound revealed another unique and more reliable display of the microvessel system. Whereas the neovascularisation of the chronic inflammatory process creates arterial and venous vessels without any signs of compression and basically in the same size, the neoplastic neovascularisation is characterized by just visible arterial microvessels without any venous microvessels visible. The method to discriminate between these kinds of vessels simply involves performing pw-Doppler scanning during the available contrast-enhancing effect of approximately 3 minutes. The fact that no venous vessels are visible in pancreatic carcinomas using contrast enhanced high mechanical Doppler endosonography means this method works even when the tumor shows an atypical rich vessel system [46].

Intraparenchymal pressure differences between pancreatic carcinoma and chronic pancreatitis might be a major cause of this unique phenomenon. This could also be indirectly shown by contrast enhanced endoscopic ultrasound. The comparison of the resistance index of arterial vessels in chronic pancreatitis to pancreatic carcinoma did show a relevant difference. In a high percentage the arterial microvessels of the pancreatic carcinoma showed a resistence index above 0.7, whereas the arterial microvessels of the areas with chronic pancreatitis showed a resistance index below 0.7. This means that neoplastic microvessels have a much higher intraluminal pressure than microvessels of the inflammatory neovascularisation [47]. It should be pointed out that the assumed difference of the intraparenchymal pressure is only a thesis which requires further studies to be backed up.

Histopathological investigations could confirm the basic pattern of microvascularisation of pancreatic carcinoma however it seems to be difficult to discriminate between arterial and venous vessels in histopathology and so no attention was given so far to this phenomenon [48].

**Figure 1:**
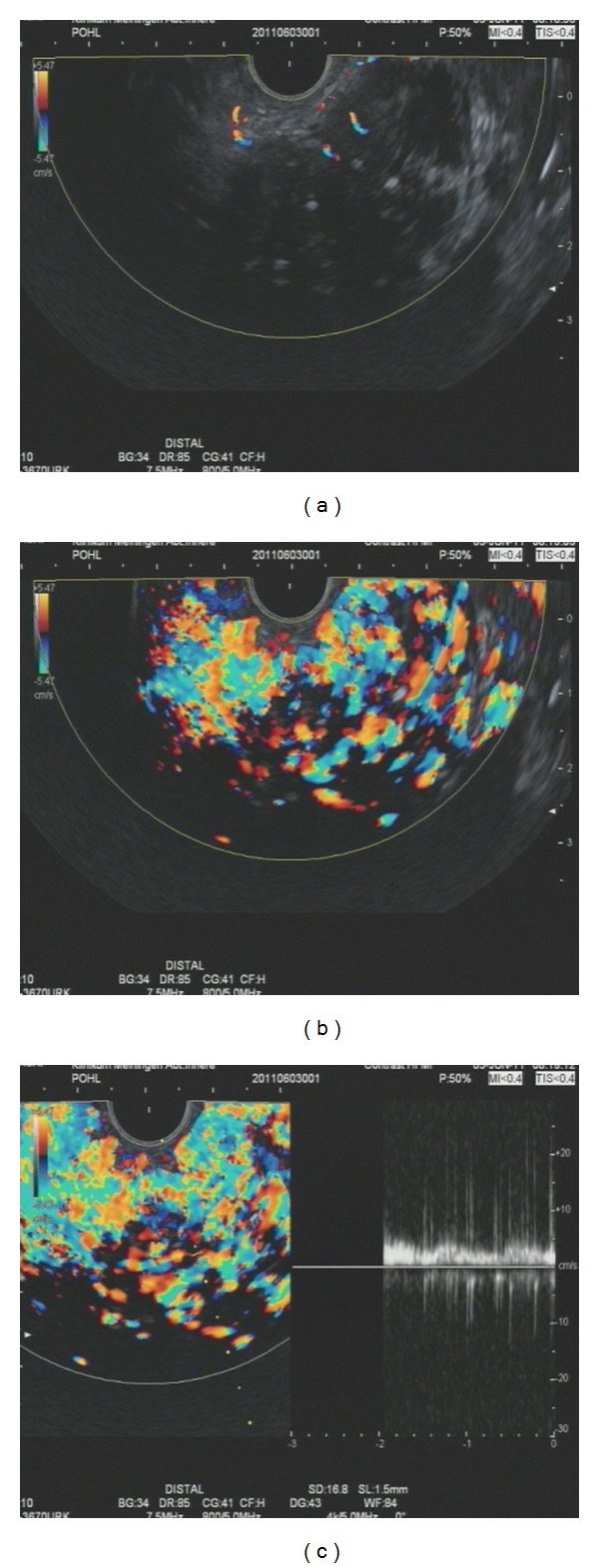
Contrast-enhanced high mechanical endoscopic ultrasound in a patient with chronic pancreatitis: (a) lesion before contrast enhancer injection the lesions is visible within the colour doppler window as a nearly black area; (b) Lesion after contrast enhancer influx (4.5 mL Sonovue) with a visible netlike vessel system the rich vessel system is visible mostly on the right side of the picture with different colours; (c) pw-Doppler analysis of the vessels with a clear venous signal; on the right half of the picture a laminar flow is displayed as a nearly flat white bark.

**Figure 2:**
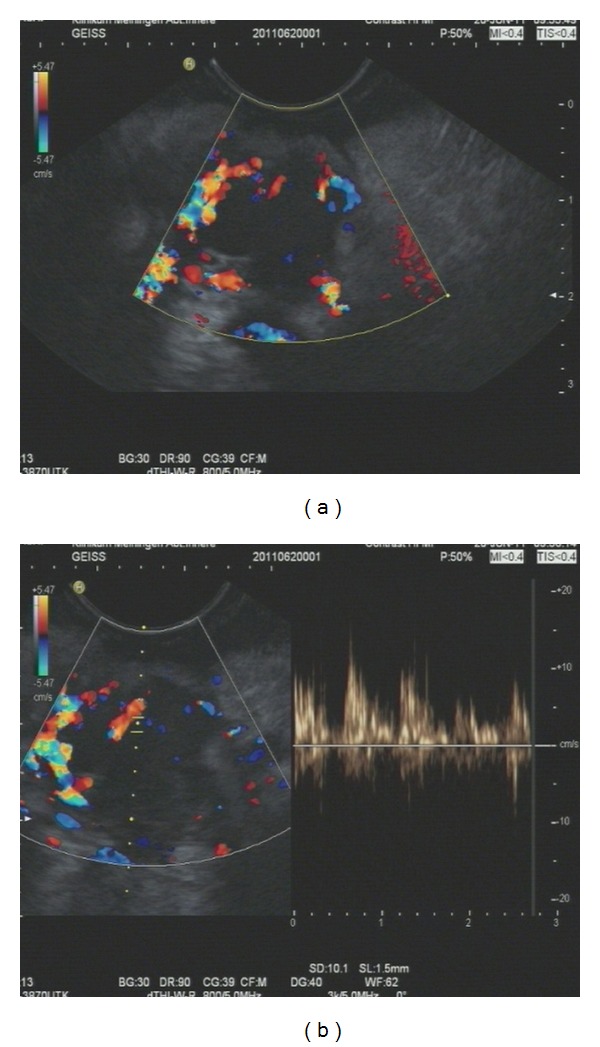
Contrast-enhanced high mechanical endoscopic ultrasound in a patient with pancreatic cancer (a) pancreatic cancer after influx of contrast enhancer (4.5 mL SonoVue); only a few vessels are visible, the lesion is visible within the colour Doppler window as the black area with the colour Doppler signals only on the edges; (b) pw-Doppler analysis reveals only arterial vessels; the atrial vessel signal appears in the right half of the picture in a pulsatile manner.

Using the method of contrast enhanced high mechanical endoscopic ultrasound with pw-Doppler vessel analysis, pancreatic carcinoma can be discriminated from chronic pancreatitis with a sensitivity and specificity over 90 percent [49] (see Figures 1 and 2).

## 4. Perfusion Studies Using Contrast-Enhanced Low Mechanical Index Endoscopic Ultrasound

**Figure 3:**
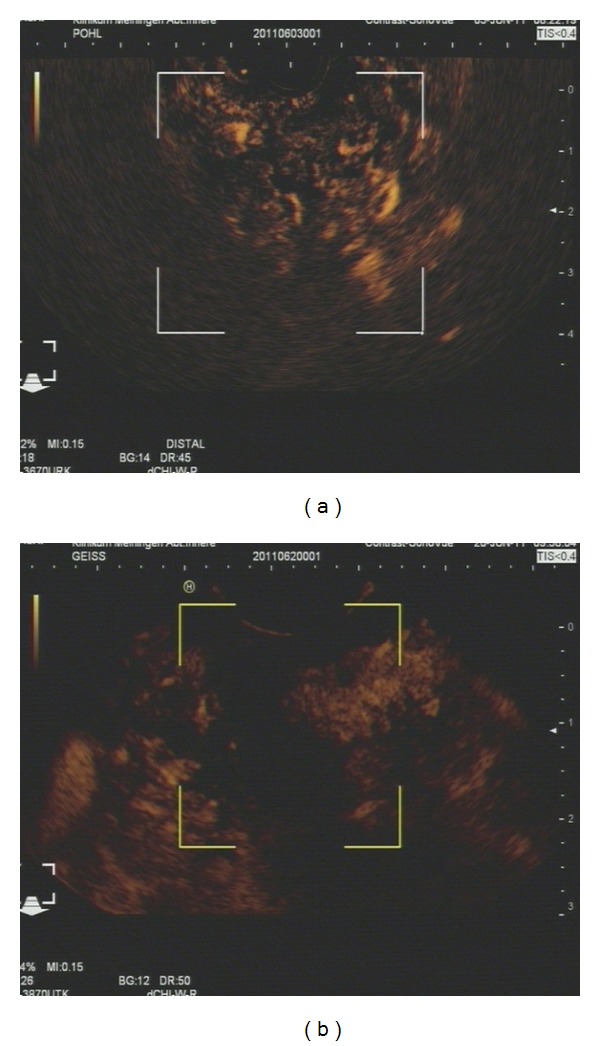
Low mechanical index contrast enhanced endosonography: (a) chronic pancreatitis with a clear enhancement of the contrast enhancer; the lesion is visible inside the markers mostly in the right upper area; all the bright visible spots are contrast enhancer signals (b) pancreatic carcinoma with a lack of contrast enhancer in the lesion; the lesion is visible within the markers; there is a black area without any contrast enhancer signals.

In 2010 contrast-enhanced low mechanical index endoscopic ultrasound evolved [50]. Because of the accuracy of the method for discriminating of liver lesions [51–54] and similarly good results for the discrimination of pancreatic lesions [55–58] in percutaneous ultrasound, there was hope that the method could increase the efficacy of endoscopic ultrasound for the discrimination of chronic pancreatitis from pancreatic carcinoma even further (see Figure 3). Initial experiences showed a reliable display of microvessel perfusions down to a size of a single contrast enhancer bubble [59–61]. However, Doppler analysis in combination with this method is not available so far and this makes the differentiation of arterial and venous microvessels impossible. Unfortunately, analyzing global perfusion behaviors of the lesions with this technique does not produce similar or better results than the method described above of contrast-enhanced high mechanical index Doppler endoscopic ultrasound [62]. This is mostly due to the fact that pancreatic lesions caused by chronic inflammatory processes often show impaired perfusion using this technique and cannot therefore be discriminated.

## 5. A Special Case: Autoimmune Pancreatitis

Autoimmune pancreatitis is a rare form of chronic pancreatitis and can involve the whole pancreatic organ as well as focal areas. Because of the diagnostic difficulties mentioned before, most of the patients are diagnosed postoperatively. Lately some patients could be diagnosed before operation because gastroenterologists' understanding of the condition has developed in the last few years.

**Figure 4:**
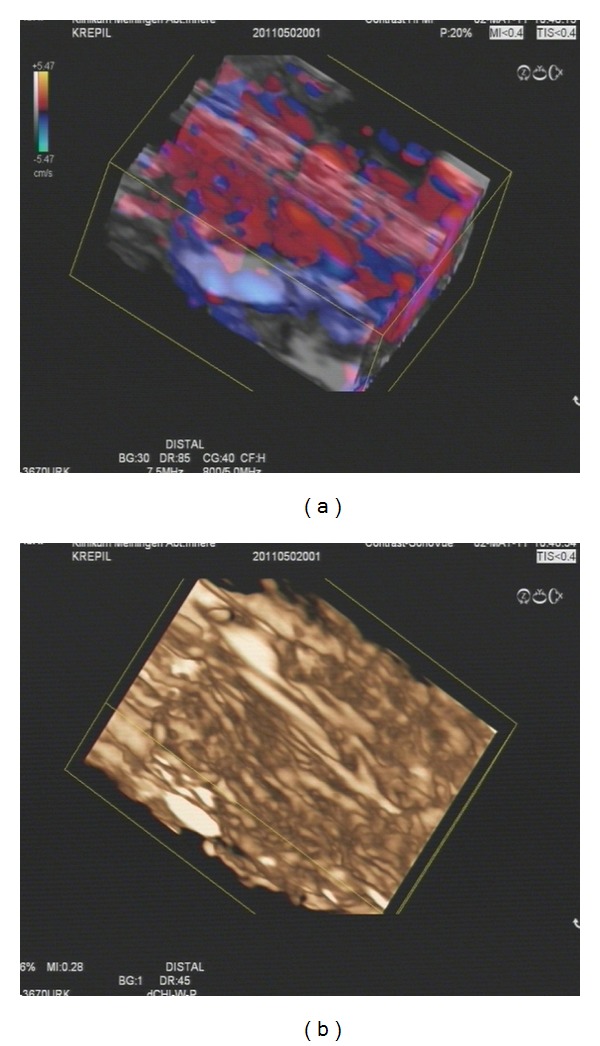
Contrast-enhanced high and low mechanical index endoscopic ultrasound with 3D reconstruction: (a) dense vessel involvement of the pancreas is impressively visible; all the red and blue spots are the Doppler colour signals from a section of the pancreas; (b) the influx of contrast enhancer is shown clearly in the low MI reconstruction to have spread homogenously through the organ.

From the CT scans it is now well known that the typical appearance of diffuse autoimmune pancreatitis is a sausage-like form of the pancreas [63, 64]. The use of contrast-enhanced high mechanical index endoscopic ultrasound as well as low mechanical index endoscopic ultrasound reveals another typical behavior [65] (see Figure 4). In patients with autoimmune pancreatitis the whole pancreatic organ shows a strong hypervascularisation as well as hyperperfusion in most cases [66]. Consequently arterial and venous vessels can be discriminated in all patients. It has to be announced that these results are only based on case studies because of the rarity of this disease, however it supports the underlying theory of different kinds of neovascularisations in chronic inflammatory processes and cancer of the pancreatic organ.

## 6. Future Developments

Analyzing different kinds of neovascularisation of chronic pancreatitis and pancreatic carcinoma and using it for diagnostic purposes in clinical practice seems to be a step forward. To further improve our diagnostic possibilities, histopathological studies investigating those results would be of special interest. In addition improving the contrast-enhanced ultrasound technique even more to identify those differences more easily, for example by using automatic analyzing systems, might be helpful in future. This is especially interesting by using contrast-enhanced low mechanical endosonography in context with perfusion studies with time-intensity-curve analysis which could be the next step to improve the technique.

As mentioned, there are different vascularisation patterns even within different pancreatic carcinomas as well as in chronic pancreatitis. Being able to relate those different types of neovascularisation to the treatment options could even improve our therapeutic options or allow us to draw prognostic conclusions [67].

## Figures and Tables

**Table 1:** Discrimination of pancreatic lesions using contrast enhancement patterns in patients with chronic pancreatitis [11].

	Number	Size of lesion (cm)	Hypervascularisation	Hypovascularisation
Chronic pancreatitis	71	3.26 ± 0.75 [1.5–4.0]	53	18
Pancreatic carcinoma	81	3.47 ± 1.04 [1.8–4.0]	24	57

## Glossary

Blooming effect: Overvisualization of the Doppler signal due to a strong signal.
Colour Doppler: Kind of an ultrasound technique to display moving particles by Doppler technique.
Low mechanical index endosonography: Pictures acquired with help of a special software of an ultrasound machine where the power of the ultrasound is reduced to a level, that the contrast enhancer bubbles remain intact and only the signals from the bubbles are displayed on the screen.
High mechanical index endosonography: Use of the contrast enhancer as an increaser of the Colour Doppler signal.
Mechanical index: Power of the ultrasound force used to create an image.
